# Incorporation of Fucoidan in β-Tricalcium phosphate-Chitosan scaffold prompts the differentiation of human bone marrow stromal cells into osteogenic lineage

**DOI:** 10.1038/srep24202

**Published:** 2016-04-12

**Authors:** Subramaniam Puvaneswary, Hanumantharao Balaji Raghavendran, Sepehr Talebian, Malliga Raman Murali, Suhaeb A Mahmod, Simmrat Singh, Tunku Kamarul

**Affiliations:** 1 Tissue Engineering Group (TEG), Department of Orthopaedic Surgery, NOCERAL, Faculty of Medicine, University of Malaya, 50603, Kuala Lumpur, Malaysia; 2 Department of Mechanical engineering, Engineering Faculty, University of Malaya, 50603 Kuala Lumpur, Malaysia

## Abstract

In our previous study, we reported the fabrication and characterization of a novel tricalcium phosphate-fucoidan-chitosan (TCP**-**Fu**-**Ch) biocomposite scaffold. However, the previous report did not show whether the biocomposite scaffold can exhibit osteogenic differentiation of human bone marrow stromal cells in osteogenic media and normal media supplemented with platelet-derived growth factor (PDGF-BB). On day 15, the release of osteocalcin, was significant in the TCP**-**Fu**-**Ch scaffold, when compared with that in the TCP-Ch scaffold, and the level of release was approximately 8 and 6 ng/ml in osteogenic and normal media supplemented with PDGF-BB, respectively. Scanning electron microscopy of the TCP-Fu-Ch scaffold demonstrated mineralization and apatite layer formation on day 14, while the addition of PDGF-BB also improved the osteogenic differentiation of the scaffold. An array of gene expression analysis demonstrated that TCP-Fu-Ch scaffold cultured in osteogenic and normal media supplemented with PDGF-BB showed significant improvement in the expression of collagen 1, Runt-related transcription factor 2, osteonectin, bone gamma-carboxyglutamate protein, alkaline phosphatase, and PPA2, but a decline in the expression of integrin. Altogether, the present study demonstrated that fucoidan-incorporated TCP-Ch scaffold could be used in the differentiation of bone marrow stromal cells and can be a potential candidate for the treatment of bone-related ailments through tissue engineering technology.

The use of metallic plates in order to support the defective region of the bone during the early stages of bone healing have a tendency to creep and deform permanently under mechanical stresses, and thus, require reinforcements. Hence, the field of tissue engineering is moving towards the development of composite scaffolds that have mechanically supportive structures that can resist creep failure whilst exhibiting osteoconductive as well as osteoinductive capabilities^1^. The present therapeutic approaches in repairing critical-size bone defects, such as those caused by tumor resection or trauma, include the use of autographs, allografts, metals, porous ceramics, and so on; however, each of these methods has their own pros and cons^2^. Since tissue engineering emerged as an alternative to overcome these limitations, particularly the structural and functional tissue in combination with scaffold and growth factors. The major aim of tissue engineering was to mimic the bone structure and good physiological function. Among various cell sources used, progenitor cells are the key for better tissue healing in combination with growth factor and scaffolding material^3^. Previous studies have shown that periosteal provides a good source of cells which can readily differentiate and lay down bone. However, the choice in most cases is bone marrow due to the limited availability of periosteal tissue^4^.

The potential of a particular material to be used as a scaffold on which stem cells can attach, proliferate and mineralize are termed as osteoconductive^5^. Though different materials have been used for bone tissue engineering, hydroxyapatite and tricalcium phosphate (TCP) are gaining in popularity as implants alone, or in combination with other components. These components enhance the active interaction with normal bone surfaces during bone healing which is vital during the regenerative process and remodeling^6^. The crystalline form of calcium phosphate being the predominant form constitutes 60–70% of bone tissue, both synthetically and naturally in TCP. TCP is a tertiary calcium phosphate also known as bone ash, a rich source of calcium and phosphorus, with the property of rapid absorption. Studies have demonstrated that β-TCP is highly biocompatible and creates a resorbable interlocking network within the defective site so as to promote healing^7^.

Chitosan is a linear, semicrystalline polysaccharide composed of (1,4)-2-acetamido-2-deoxy-b-D-glucan (N-acetyl D-glucosamine) and (1,4)-2-amino-2-deoxyb- D-glucan (D-glucosamine) units. Chitosan has been proved to have various intrinsic properties like antibacterial, antifungal, mucoadhesive, analgesic, hemostatic and degraded as nontoxic resides^8^. The rate of degradation is related to the molecular mass of the polymer, and has some extent of biocompatibility and physiological medium. It has been shown that the presence of a positive charge on Chitosan can act with negatively charged blood cells in cell membranes^9^.

Fucoidan, a sulfate-rich polysaccharide, is an anionic type comprised of fucose, and is a good candidate for reinforcing the component of cellular activities for bone regeneration. Reports have shown that addition of fucoidan to the cultures can induce fibroblast growth factor-2 activity, assist fibrillar collagen matrix formation and enhance fibroblastic proliferation. There are studies demonstrating the incorporation of fucoidan with polycprolactone. Likewise, fucoidan significantly induced osteogenic differentiation of hADSCs, increasing expression of osteogenic marker genes, including alkaline phosphatase (ALP), collagen 1 (Col1), osteonectin (ON), and Runt-related transcription factor 2 (Runx2). hADSCs are obtained from adipose tissue. Studies have also demonstrated that fucoidan in a 3D scaffold interacts with vascular endothelial growth factor and promotes neovascularization in mice^10^,^11^,^12^. *In vitro* studies using MG-63 cells showed fucoidan in chitosan alginate scaffold improved cell proliferation and release of ALP. In addition, fucoidan has the ability to bind to growth factor via its heparin-binding domain^13^. The formation of an adherent hematopoietic culture in the presence of fucoidan was mediated via cell adhesion factors like L-selectin and integrin. Platelet-derived growth factor (PDGF-BB) has been known to have potential to induce the proliferation and migration of the stromal cells. However, contrary reports that exist could impede the differentiation process while some reports indicated it could enhance differentiation^14^. On the other hand, microsphere-loaded PDGF-BB improved wound healing *in vivo*. Having reported on fabrication and characterization of the scaffold in our previous study, we have tested the potential of a scaffold in inducing differentiation of human bone marrow stromal cells (hMSCs) with or without PDGF-BB, with reference to osteogenic marker, biomineralization changes, and gene expression^15^.

## Results

### Optimization of fucoidan percentage in a TCP-Ch scaffold

**Figure 1:**
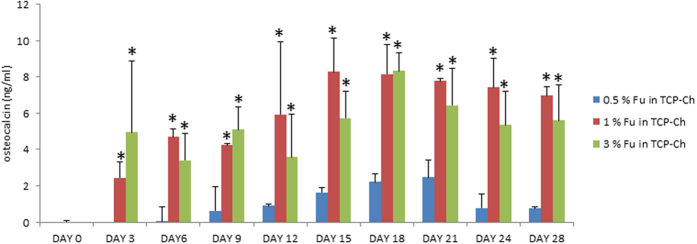
The OC levels in the TCP-Ch (supplemented with 0.5%, 1%, and 3% fucoidan) scaffolds at variable time points for 28 days. The OC released at different points in time was quantified using the commercially available ELISA kit. The data were represented as the means ± Standard deviation (SD). The statistical significant was set at level P < 0.05, post-hoc followed by the least significant deviation (LSD) which was performed using SPSS version 10. ^*^Represents the TCP-Ch containing different percentage of Fu. at day variable points in time.

Choice of TCP-Fu-Ch was based on the preliminary analysis of different concentrations of fucoidan (0.5, 1 and 3%) incorporated in a TCP-Ch composite. TCP-Fu-Ch seeded with hMSCs was cultured in commercial osteogenic media, and the release of osteogenic marker osteocalcin (OC) was monitored at different time points, as shown in Fig. 1. It was observed that the level of OC release in 1% and 3% fucoidan-incorporated TCP-Ch scaffold was significant (p < 0.05), when compared with that in 0.5% fucoidan-containing scaffold. Although the release rate in 1% and 3% fucoidan-containing TCP-Ch scaffold was similar, we selected 1% fucoidan-containing scaffold for further experiments, because the 3% fucoidan-containing TCP-Ch scaffold was fragile toward the end time point of analysis.

### Apatite formation on scaffolds immersed in SBF

**Figure 2:**
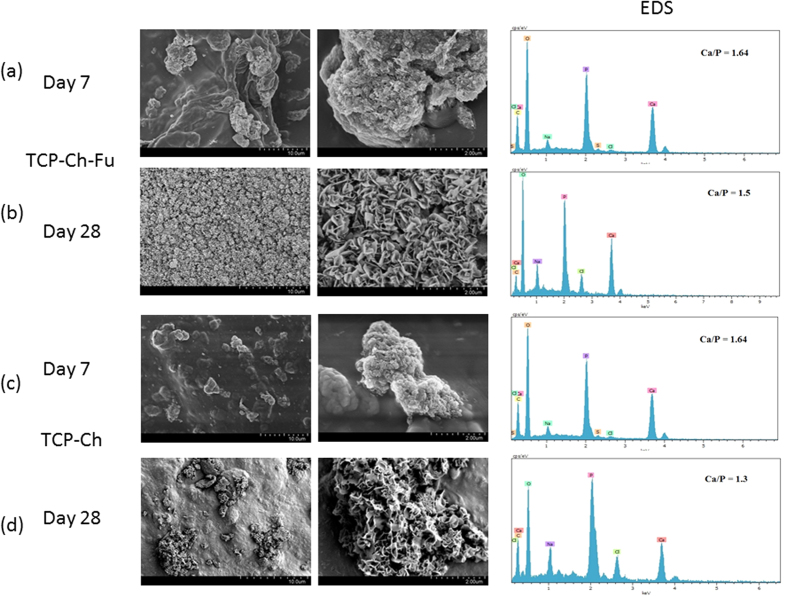
(**a–d**) Results of apatite formation and EDX analysis for the TCP-Fu-Ch and TCP-Ch scaffolds immersed in SBF. For FE-SEM assessment, the scaffolds were sputter-coated with platinum and an accelerating voltage of 2 kV and a working distance of 13.9–16.9 mm were employed. EDX analysis was performed using the EDX System (S-4800, Hitachi, Japan) attached to the FE-SEM instrument with an accelerating voltage of 10 kV.

The FE-SEM and EDX results were collectively used to evaluate the response of the scaffolds after immersion in SBF for 7 and 28 days. Figure 2a,d shows the surface morphology and corresponding EDX spectra of the TCP-Ch and TCP-Fu-Ch scaffolds after immersion in SBF for 7 days. It can be observed that both the scaffolds exhibited mineral-like precipitates in the shape of bundles of aggregates with packed-rod-like morphology at higher magnifications. However, the TCP-Fu-Ch scaffolds showed more deposition of the mineral layer on their surface, when compared with the TCP-Ch scaffolds. Furthermore, the EDX results of both the scaffolds after 7 days of immersion in SBF revealed the presence of calcium and phosphorous on the surface of the deposited layer with a Ca/P ratio of 1.64 (for both the scaffolds), which was nearly close to that of stoichiometric hydroxyapatite (1.67). Figures 2c,d illustrate the morphology of the deposited layer and the corresponding EDX spectra of the TCP-Ch and TCP-Fu-Ch scaffolds, respectively, after 28 days of immersion in SBF. Interestingly, the surface morphology of the deposited layer on the TCP-Ch scaffolds remarkably differed from that of the TCP-Fu-Ch scaffolds after 28 days of immersion in SBF. The deposited layer on the TCP-Ch scaffolds contained some bundles of dense plate-like crystals (with sharp edges of about 30-nm thickness and 200-nm width) developed perpendicular to the surface of the scaffold, covering a small portion of the surface and indicating poor apatite-forming capability. Conversely, the deposited mineral layer on the TCP-Fu-Ch scaffolds consisted of aggregated flower-like particles (which are typical for bone-like apatite) with a mean diameter of 800 nm, covering the entire surface of the scaffold and indicating superior apatite-forming capability. The higher resolution FE-SEM images revealed that these flower-like mineral depositions consisted of tiny plate-like crystals with a thickness of nearly 20 nm. In addition, the EDX results of the TCP-Ch and TCP-Fu-Ch scaffolds after 28 days of immersion in SBF showed obvious differences in the Ca/P ratio, which was 1.3 and 1.5, respectively. These findings indicated that the apatite layer formed on the composites was calcium-deficient carbonated apatite with a Ca/P ratio of less than 1.67, which is usually noted in the latter days of immersion in SBF because the calcium-rich amorphous calcium phosphate (ACP) on the scaffold specifically interacts with negative phosphate ions in the SBF to form calcium-deficient ACP.

### Cell viability

**Figure 3:**
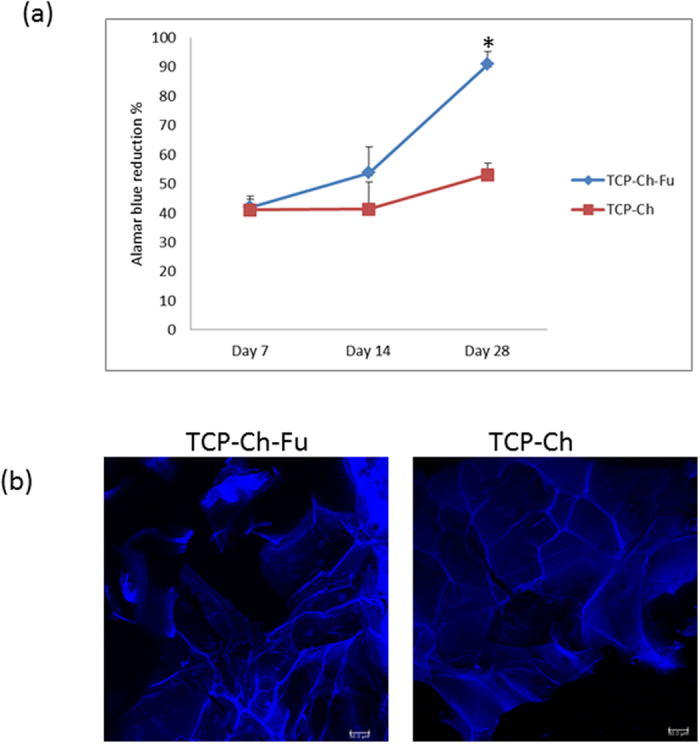
(**a**) Results of Alamar Blue reduction (%) for the TCP-Fu-Ch and TCP-Ch scaffolds at different time points (7, 14, and 28 days). Alamar Blue working solution was added to the test and control wells, followed by incubation for 4 h and measurement of absorbance at 570 and 600 nm. 3 (**b**) Confocal microscopy images of the TCP-Fu-Ch and TCP-Ch scaffolds seeded with mesenchymal stromal cells. The blue stained regions indicate attachment of the cells to the scaffold.

At 28 days, the cell viability of the TCP-Fu-Ch scaffold was considerably significant (p < 0.05), when compared with that of the TCP-Ch scaffold; however, no significant difference was noted at the early time points (7 and 14 days) (Fig. 3a). Furthermore, confocal microscopy results confirmed the cell viability of both the scaffolds (Fig. 3b).

### Osteocalcin release

**Figure 4:**
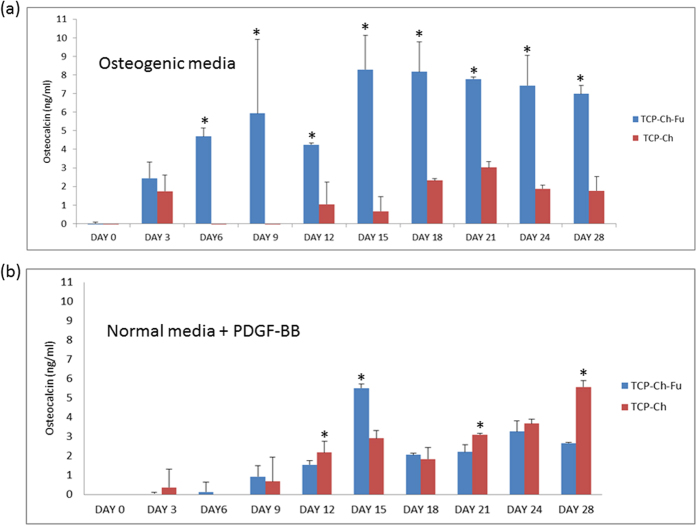
(**a,b**) The OC levels in the TCP-Fu-Ch and TCP-Ch on every 3 days (0 to 28 days) in osteogenic media and normal media supplemented with PDGF-BB. The OC released at different points in time was quantified using the commercially available ELISA kit. The data were represented as the means ± Standard deviation (SD). The statistical significant was set at level P < 0.05, post-hoc followed by the least significant deviation (LSD) which was performed using SPSS version 10. ^*^Represents the TCP-Fu-Ch compared with the TCP-Ch at day variable points in time.

The level of OC release in the TCP-Fu-Ch scaffold seeded with hMSCs was significant (*p* < 0.05), when compared with that in the TCP-Ch scaffolds (in osteogenic and normal media with PDGF-BB; Fig. 4a,b). From day 15 to day 28, a four-fold increase was noted, while addition of PDGF-BB with normal media rapidly increased OC on day 15, and OC levels in TCP-Fu-Ch was increased by threefold when compared with TCP-Ch followed by a decrease. The increase in OC in TCP-Ch was observed on day 28 (*p* < 0.05).

### Scanning electron microscopy of TCP-Fu-Ch and TCP-Ch

**Figure 5:**
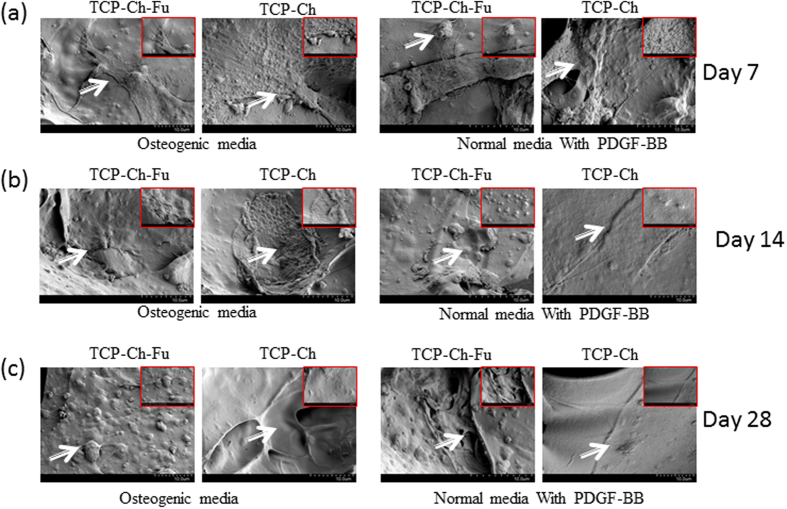
(**a–c**) SEM of the TCP-Fu-Ch and TCP-Ch at different points in time (7, 14, and 28 days). Mesenchymal stromal cells were cultured on these scaffolds and subjected to the CPD procedure. Following the CPD procedure, the samples were viewed under SEM (Phenom G2 Pro equipped with Fiber metric-Pro-Suite application). Arrows indicate topographical appearance of fibrous substrate and calcium-like apatite of hMSCs cultured with osteogenic media and normal media with PDGF-BB supplement.

Surface topographical features of cells on scaffold were observed with scanning electron microscopy at days 7, 14 and 28 (Fig. 5a–c). After day 7 of culture, some cells appear flatly on the surface of the scaffold with PDGF-BB, while the PDGF treatment showed some matrix-like formation with some cells flatly, and some spindle-shaped cells are visible in both types of scaffolds. The elongation of the cells was considerable in both the scaffolds. The cell-to-cell contact and attachment was observed on day 7. On day 14, the cells on TCP-Fu-Ch and TCP-Ch appeared flatly, and spreading was uniform on the scaffold. Supplementation of PDGF-BB induced layer formation and was well spread on the scaffold and on some lamellipodia. On day 28, a dense and continuous layer of cell sheet was formed on the scaffolds on both the TCP-Fu-Ch and TCP-Ch with or without growth factor supplementation. While mineralization was considerably better in TCP-Fu-Ch when compared with the TCP-Ch. Addition of growth factor improved the mineralization in TCP-Fu-Ch compared to TCP-Ch surfaces. Overall addition of growth factor improved spherical and polygonal morphologies attached to the surface and around the pores, and exhibited fusiform spherical morphologies.

### Gene expression TCP-Fu-Ch and TCP-Ch

**Figure 6:**
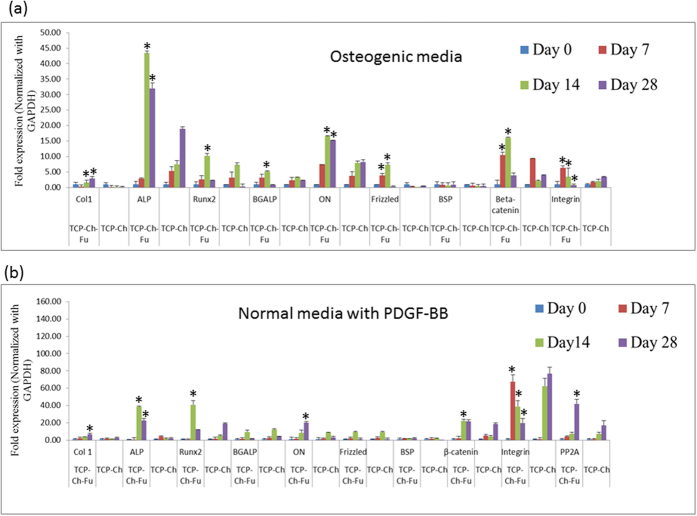
(**a,b**) Quantitative expression of Col1, ALP, Runx2, BGALP, ON, Frizzled, BSP, β-catenin, integrin, and PP2A in the TCP-Fu-Ch and TCP-Ch scaffolds during cell differentiation (in osteogenic and normal media supplemented with PDGF-BB). The total RNA was extracted from hMSCs cultured on the substrates (n = 6) at day 0, 7, 14 and 28 using the RNeasy Mini Kit (Qiagen, Chatsworth, CA, USA). Following the cDNA synthesis and qPCR, the relative gene expression was normalized to the GAPDH and baseline expression. The data were represented as the means ± Standard deviation (SD). The statistical significant was set at level P < 0.05, post-hoc followed by the least significant deviation (LSD) which was performed using the SPSS version 10. ^*^Represents the TCP-Fu-Ch compared with the TCP-Ch on all time points.

We examined an array of genes related to osteogenesis, the genes Col1, ALP, Runx2, OC, bone gamma-carboxyglutamate (Gla) protein (BGALP), Osteonectin (ON), Frizzled, bone sialo protein (BSP), beta-catenin (β-catenin), integrin, and PP2A were examined at different time points (on days 0, 7, 14, and 28) in both the scaffold cultured in osteogenic media and normal media supplemented with PDGF-BB (Fig. 6a,b). On day 14, the expression of Col1, ALP, Runx2, BGALP, and ON was significantly increased (*p* < 0.05) in the TCP-Fu-Ch scaffold, when compared with that in the TCP-Ch scaffold. Furthermore, the levels of Frizzled, β-catenin, and integrin were high in the TCP-Fu-Ch scaffold on days 7 and 14, and subsequently declined on day 28. In addition, the TCP-Fu-Ch scaffold also showed increased expression of the osteogenic genes when growth factor was supplemented. The expression of Col1, ALP, Runx2, ON, and β-catenin increased in the TCP-Fu-Ch scaffold on days 7, 14, and 28, when compared with that in the TCP-Ch scaffold (*p* < 0.05). β-catenin was significantly high on days 14, while integrin was high on day 7, and in TCP-Fu-Ch was declining gradually at the 14 and 28 day points. In addition, the gene, PP2A, increased at day 28 in TCP-Fu-Ch when compared with TCP-Ch (*p* < 0.05).

## Discussion

The porous scaffold can be considered as a suitable candidate for providing the physiological environment for cells to contact each other with surplus nutrient transfers. Some previous studies have reported that distinct nodules were visibly loaded with stem cells. It has also been found that the presence of factors, such as calcium related to osteogenic differentiation, showed deposition of calcium phosphate inside the matrix^16^. In the present study, we demonstrated similar results by observing areas of mineralization and apatite formation using scanning electron microscopy and a simulated body fluid method. The formation of a layer-like structure and mineralization in the TCP-Fu-Ch scaffold indicated that it could promote early cell proliferation and differentiation. Although a major difference was not observed when compared with TCP-Ch in inducing mineralization, addition of PDGF-BB showed some difference in the pattern of nodules on both the scaffolds. The observed calcified nodules were formed within the pores of the scaffolds, indicating that the physiological environment was suitable for the cells to attach, proliferate and differentiate. These results also indicate that the cell-to-matrix interaction was considerably better.

The formation of bone is a dynamic process that needs a thorough understanding of how bone cells interact with the matrix. The results of the present study demonstrated that the 3D scaffold seeded with stromal cells induced early differentiation of cells to form bone-like cells. OC is the non-collagenous protein of the bone. OC binds to the calcium and plays an important role during the mineralization which is a precondition for osteoblastic differentiation. An increase in the levels of OC indicates early osteogenic differentiation of stromal cells seeded on a TCP-Fu-Ch scaffold^17^. Although certain level of OC was noted in the TCP-Ch scaffold, the release was delayed, indicating that cell differentiation was not rapid, when compared with that in the TCP-Fu-Ch scaffold. Moreover, addition of PDGF-BB to normal medium improved the release of OC at an early time point in the TCP-Fu-Ch scaffold, when compared with that in the TCP-Ch scaffold. These findings demonstrated that the TCP-Fu-Ch scaffold could induce cell differentiation regardless of the culture medium (osteogenic or normal medium supplemented with PDGF-BB). The cell viability was confirmed using Alamar Blue assay, which revealed significantly higher cell viability for the TCP-Fu-Ch scaffold, when compared with that for the TCP-Ch scaffold. Furthermore, confocal microscopy results established the infiltration and attachment of cells onto the scaffold.

The bone bonding ability and osteointegration (*in vivo* bioactivity) of a scaffold are occasionally predicted by its capability to form an apatite layer on its surface *in vitro* upon immersion in SBF (except in special cases, where the scaffold can be integrated within the host bone tissue without detection of a visible apatite layer on their surface)^18^,^19^. Interestingly, the overall findings of the present study conform, both quantitatively (Ca/P ratio) and qualitatively (morphology), to those reported previously with regard to apatite formation after the initial stages of immersion in SBF^20^,^21^. The calcium-deficient ACP is known as octacalcium phosphate (OCP), a precursor of hydroxy apatite HA, which later crystallizes into bone-like apatite upon equilibrium^22^,^23^. Accordingly, the Ca/P ratio of the apatite formed on the TCP-Ch composite was 1.3, which is closer to the OCP ratio (1.33), whereas that of the apatite formed on the TCP-Fu-Ch scaffold was 1.5, indicating formation of an intermediate phase containing a combination of OCP and HA. It must be noted that OCP is most likely to form than HA on TCP ceramics immersed in SBF *in vitro*.

Though many studies have reported on different composite materials with osteogenic supplement, the growth factors are essential for cellular signaling for migration, proliferation and differentiation of stromal cells into osteogenic-like cells^24^. PDGF-BB composed of A, B, C and D polypeptide chains not only participates in embryonic development of major tissues like kidney and heart, but also in regeneration. Studies have shown that PDGF can improve chemotaxis, mitogenesis, proliferation and matrix synthesis, and the Food & Drug Administration has approved the promoting of bone repair in periodontal osseous defects^25^. The regulatory mechanism of stromal cell differentiation remains to be elucidated. Reports have shown that the application of PDGF-BB enhances bone formation, whereas one report has shown that it does not play a role in bone formation^26^,^27^,^28^. Though the role of PDGF-BB in bone formation *in vitro* is still unclear with conflicting reports, one of our previous data on the use of PDGF-BB induces partial differentiation of stromal cells on nanofibrous material under normal media conditions^29^. The current evidence suggests that the osteogenic potential does depend on the support scaffold and type of growth factors synergistically.

In bone, TCP-based scaffolds haven been used to provide a local environment for cell attachment and support matrix for direct cellular growth which supports bone remodeling. Most of the polymer-based scaffolds are able to guide the reparative growth of the natural bone, but do not possess the ability to induce differentiation of precursor cells into bone-like cells^30^. Our results show that a combination of TCP-Fu-Ch induced differentiation of hMSCs when compared with TCP-Ch. Scaffold design considerations include surface chemistry for guiding cellular behavior and integration with host tissue. Recently, the effectiveness of implant integration with host defect has been improved to allow better osseous tissue in growth, thus promoting long-term implantation^31^. In order to improve on this, appropriate bioactive materials, such as TCP-based compounds into chitosan and fucoidan could be helpful in creating a bioactive composite. These bioactive materials have the ability to form strong bone with bone when placed in the vicinity of host bone^10^.

The ALP and OC gene expressions are usually related to the early differentiation of the hMSCs scaffold culture. We have demonstrated that an increase in OC correlated approximately with osteogenic media supplement (*p* < 0.05), while an increase was noted in both the scaffolds after PDGF-BB supplementation, while statistically no significance was observed. These results demonstrate that mineralization is a slow process and adequate time is required for complete mineralization in case of single growth factor, while osteogenic media induced early differentiation. TCP-Fu-Ch showed an approximately two fold increase in Runx2 compared with (*p* < 0.05) TCP-Ch. Runx2 is a zinc finger transcription factor, which is essential for osteoblast differentiation, acting on the downstream Runx2, and modulating the expression of important osteoblast proteins, such as osteopontin, ON, bone sialoprotein, and collagen type I. Osteopontin and ON are phosphoproteins containing several Ca-binding domains expressed in the differentiating osteoblasts. It regulates cell adhesion, proliferation, and ECM mineralization during bone development^32^. Biocomposite multilayer biomimetic scaffolds can mimic the osteocartilaginous structure, and are well suited to culture stromal cells to differentiate into osteogenic-like cells. The TCP-Fu-Ch environment provides the surface for cell attachment, and enhances the proliferation of cells by using mitogens, such as PDGF-BB for rapid differentiation of the stromal cells. The TCP-Fu-Ch surface also enhances the cell matrix interactions and cell–cell communications, which are important for the exchange of oxygen and nutrients between cells^33^. In this study, the increase noted in Col1 in TCP-Fu-Ch is compared with the TCP-Ch scaffold. Col1 is necessary, for osteogenesis stimulates the preosteoblast cell surface integrins leading to the activation of other core-binding factors, and increases the level of surface integrin^34^. Similarly, the integrin levels also significantly increased at an early time point and declined at a later time point. Previous studies have demonstrated that integrin is required for the differentiation of osteoblast cells. In general, integrin recognizes the cell adhesion molecule counter receptor on endothelial cells and extracellular matrix proteins such as fibronectin and collagen^35^. In our previous study, PDGF-BB-treated cells on nanofibrous scaffold showed increased expression of integrin at an early time point and declined expression at a later time point^36^. The findings of the present study suggest that the differentiation of cells was rapid on the TCP-Fu-Ch scaffold, while it was not significant and rapid on the TCP-Ch scaffold. It must be noted that the correlation between integrin expression and hMSC differentiation with respect to their interactions with the biomaterial surfaces has not been well investigated. Previous studies have shown that the changes in the cell shape or integrin expression could regulate the commitment of mesenchymal cells to different lineage^37^,^38^. It has been established that integrins are the links between cells and ECM and serve as signal transducers regulating cell growth, differentiation, and motility^39^,^40^. However, blocking of α5β1 integrin using antibodies did not affect the differentiation of cultured osteoblasts, suggesting that an alternate integrin partner may be involved^41^,^42^.

ON is a noncollagenous component of the ECM and is considered bone-specific because of its biochemical properties, such as a marker related to osteoblastic functional differentiation^43^. When TCP-Fu-Ch was supplemented with PDGF-BB, it showed increased ON levels (*p* < 0.05). However, there was an increase in the ON levels with the TCP-Ch scaffolds, but were not less. Previous reports have shown that ON is observed in the petri dish during osteogenic differentiation with or without ECM. When the stromal cells were cultivated in the petri dish without the ECM, the ON level was relatively less during the early stages of differentiation, but increased after the addition of the osteogenic medium after one week^44^. The increase in the ON on days 14 and 28 in the TCP-Fu-Ch scaffolds indicates that osteogenic media was more supportive at the beginning of differentiation. Supplementation with PDGF-BB does not influence the increase in the osteogenic differentiation in the early time point which, however, improves on day 28 in TCP-Fu-Ch. BGALP or OCN, are post proliferative osteoblast producers, enhancing the differentiation progress of the hMSCs biocomposite scaffold^45^.

Bone formation is a highly complex process that is organized by various growth mediators and cells. Tissue engineering mimics this regenerative process using exogenous or endogenous growth factors with scaffolds. In our previous research, we have shown that fibrous scaffolds can induce osteogenic differentiation^29^. In this study, we hypothesized that the introduction of a single mitogen like PDGF-BB would speed up osteogenic differentiation using stromal cells compared to osteogenic media. Previous studies have shown that stem cell transplantation with a scaffold like HA/TCP would be more effective than the cell-free scaffold in animal models^46^. Similarly, a combined therapy of bone morphogenic protein 2 with bone graft materials promotes bone regeneration. The increased production of BSP considerably enhances osteogenic differentiation^47^. In the present study, an increase in the BSP was not significant in both the scaffold supplemented with osteogenic media and normal media with PDGF-BB (*p* < 0.05). Biomaterials exhibit bone induction through the intramembranous ossification process, whereas growth factors induce bone formation through endochondral ossification^47^. Although supplementation with PDGF-BB induced differentiation of the stromal cells in TCP-Fu-Ch, this effect was not seen in TCP-Ch. This could be due to the differences in composition, especially the absence of fucoidan. Reports have demonstrated that β-catenin promotes osteogenic differentiation *in vitro*. By contrast, some reports have indicated β-catenin signaling inhibits the osteogenic differentiation and mineralization process^48^. In the present study, the TCP-Fu-Ch scaffold showed significant increase at early time points 7 and 14 (*p* < 0.05), while a decrease in β-catenin was observed on day 28. A similar trend was observed in the TCP-Ch at an early time point. No increase was observed in the β-catenin level which was noted after PDGF-BB supplementation at an early time point 7, while high levels were noted on day 28 in TCP-Fu-Ch in TCP-Ch. These changes in the β-catenin levels indicated the balance in MSC differentiation, and the fact that although β-catenin is both osteoinhibitory and inductive, it is also time-dependent. Previous studies have demonstrated that basal Frizzled mRNA levels also increase when primary hMSCs differentiate into osteoblasts, reinforcing the developmental regulation of the gene^49^. Accordingly, in the present study, the expression of frizzled gene was significant on days 7 and 14 in the TCP-Fu-Ch scaffold cultured in osteogenic medium, whereas no significant changes were observed in the TCP-Fu-Ch and TCP-Ch scaffolds cultured in normal medium with PDGF-BB supplementation. As the frizzled signaling pathways are known to be intimately associated with differentiation, cell fate, and early development, the finding of the present study suggests that Frizzled is one of the factors in activation and proliferation^50^. The PP2A (serine/threonine phosphatase) regulates diverse cellular functions, such as transcription and signaling. One of the major functions of PP2A is to regulate the mitogen-activated protein kinase-related pathway^51^. Studies have shown that compounds and molecules modulating PP2A may be used for treating osteoporosis. One study has also demonstrated that PP2A has a main role in controlling differentiation of hMSCs^52^. In our study, surprisingly, the PP2A was expressed in the group of scaffold TCP-Fu-Ch treated with PDGF-BB but not in the scaffold TCP-Ch. These results are in accordance with a previous report that PDGF-BB may have some role in inducing PP2A.

## Conclusion

TCP-Fu-Ch can trigger differentiation of hMSCs into osteogenic-like cells when compared with TCP-Ch when cultured in osteogenic media and normal media supplemented with PDGF-BB. Hence, this incorporation of fucoidan in TCP-Ch can serve as a potential candidate for bone-related tissue engineering applications.

## Methods

### Human bone marrow stromal cell culture

Human bone marrow was harvested from adult donors undergoing intramedullary nailing at the University of Malaya Medical Center after obtaining written informed consent. The methods were followed in accordance with the approved guidelines. All the experimental protocols were approved by the medical ethics committee (Approval ID No. MECID.NO: 201412-859, UMMC). A total of 2 ml of bone marrow was diluted with 2 ml of phosphate-buffered saline (PBS; pH 7.2) and layered onto 3 ml of Ficoll-Paque Premium (GE Healthcare, Sweden) before subjecting to gradient centrifugation at 1800 rpm for 30 min (Eppendorf 5810R). The mononuclear layer was then collected and washed twice with low-glucose Dulbecco’s modified Eagle’s medium (DMEM) (Invitrogen-Gibco, USA) supplemented with 1% antibiotic/antimycotic (v/v; Invitrogen-Gibco). The isolated mononuclear cells were cultured in a growth medium (low-glucose DMEM supplemented with 10% fetal bovine serum, 1% antibiotic/antimycotic (v/v), and 200 mM GlutaMAX TM-I (Invitrogen-Gibco)) and transferred into T75 tissue culture flasks (Nunc TM, USA). The medium was changed on day 5 to remove non-adherent cells, and subsequent medium replacement was conducted at 3-day intervals. The surface markers were examined using a flow cytometer to confirm the characteristics of mesenchymal stromal cells (Supplementary data).

### Cell seeding on the scaffolds

The isolated cells were maintained in continuous cultures without recryopreservation until they reached a predetermined number of passages. Prior to cell seeding, the scaffolds were sterilized using 70% ethanol for 20–30 min and rinsed thrice in PBS and twice in the growth medium. Subsequently, the hMSCs were detached until passage 3 and seeded onto the scaffolds in 6-well plates with a cell density of 10^4^ or 10^5^ cells/cm^2^. The medium was changed at predetermined time points (3-day interval). The samples were collected and the cells were treated with varying concentrations of PDGF-BB (25, 50, and 100 ng/ml), including one minimal optimal concentration of PDGF-BB (50 ng/ml), which induced significant release of osteogenic markers and proliferation.

### *In vitro* apatite formation

Biomineralization of the scaffolds was evaluated by examining the capability of the scaffolds to form a bone-like apatite layer on their surface upon immersion in simulated body fluid (SBF), which was prepared according to a previously described method^21^. The samples were immersed in SBF for 7 and 28 days, and the SBF was replaced every 2 days. After predetermined soaking time points, the samples were removed from the SBF solution and gently washed with double distilled water and dried at room temperature. The surface of the SBF-immersed samples was assessed using a field emission scanning electron microscope (FE-SEM) (FE-SEM; High-resolution FEI Quanta 200F, Hitachi, Japan). Prior to FE-SEM assessment, the samples were sputter-coated with platinum and an accelerating voltage of 2 kV and a working distance of 13.9–16.9 mm were employed to view the samples. Furthermore, to study the elemental composition of the apatite layer formed on the scaffolds, energy-dispersive X-ray (EDX) analysis was performed using the EDX System (S-4800, Hitachi, Japan) attached to the FE-SEM instrument with an accelerating voltage of 10 kV.

### Cell topographical analysis

In order to observe the cells adhering to the sample surface after culturing at variable time points by SEM, the postfixed scaffolds (2.5% formalin) were dehydrated with a series of graded ethanol–water solutions (10–100%), and kept in a fume hood to dry at the ambient temperature. Scaffolds were dried, sputter-coated with platinum, and observed. The resulting scaffold materials were analyzed using SEM (Phenom G2 Pro equipped with Fiber metric-Pro-Suite application) to determine the diameter and its pattern of cell distribution.

### Cell viability assay

About 0.25 ml of Alamar Blue working solution was added to the test samples and control wells, followed by incubation for 4 h and measurement of absorbance at 570 and 600 nm. The scaffolds seeded with bone marrow stromal cells were stained with Hoechst 33342 blue (Invitrogen, USA) and analyzed using a confocal microscope (Nikon C-HGFI, Japan) after 20 min of incubation at room temperature.

### Osteocalcin assay

OC assay was performed by using a commercial ELISA kit (IBL International, Germany). Concisely, monoclonal antibody was directed against epitopes of human OC. The calibrators and samples reacted with the captured monoclonal antibody coated onto the microtiter wells and labeled with a monoclonal antibody and horse radish peroxidase. After the incubation period which allowed formation of the sandwich, the plates were washed. Chromogenic solution was added to the plates and the plates were incubated and the substrate turnover was read at 450 nm with a 96-well plate reader (Biotek, USA).

### Real-time PCR

To quantify the levels of gene expression, the total RNA was extracted from hMSCs cultured on the substrates (n = 5) at different time points (0, 7, 14 and 28 days) using the RNeasy Mini kit (Qiagen, Chatsworth, CA, USA). The concentration of the harvested RNA was determined by measuring the absorbance at 260 nm using a nanophotometer (Implen, Germany). The synthesis of cDNA was with 25 ng of pure RNA using the Superscript III First Strand Synthesis Kit, according to the manufacturer’s instructions. To examine the extent of osteogenic differentiation, quantitative real-time PCR (qRT-PCR) was performed using a StepOnePlus Real-Time PCR System (Applied Biosystems, Foster City, CA, USA) with SYBR green qPCR gene expression assays for osteogenic and chondrogenic genes. The relative expression levels of the target genes were determined using the comparative Ct method, by normalization to the endogenous reference (glyceraldehyde 3-phosphate dehydrogenase (GAPDH). The relative gene expression involved in osteogenic differentiation of hMSCs cultured on each substrate was normalized to osteogenesis markers in hMSCs cultured on the control substrate. The forward and reverse primers used for the experiment are shown in Table 1.

### Statistical Analysis

The values obtained were averaged and expressed as means ± standard deviation (SD). Statistical differences were determined using SPSS Version 10, post-hoc analysis, followed by ANOVA and LSD. The differences were considered statistically significant if the value of *p* was <0.05.

## Additional Information

**How to cite this article**: Puvaneswary, S. *et al*. Incorporation of Fucoidan in β-Tricalcium phosphate-Chitosan scaffold prompts the differentiation of human bone marrow stromal cells into osteogenic lineage. *Sci. Rep*. **6**, 24202; doi: 10.1038/srep24202 (2016).

## Supplementary Material

Supplementary Information

## Figures and Tables

**Table 1:** Forward and reverse primers.

Name	Sequence	Length
β-catenin F	TGTGGTCACCTGTGCAGCTGGA	22
β-catenin R	TGGCAGGCTCAGTGATGTCTTCC	23
Frizzled F	TGGCGCTCAGCTCGGTGGAC	20
Frizzled R	AGCGGATGCGGAAGAGGGACAC	22
PP2A F	GAGGGCCCAATGTGTGATCTGTT	23
PP2A R	CAAGCTGGTGGGCACGAGAAA	21
Col 1 F	CCCGCAGGCTCCTCCCAG	18
Col 1R	AAGCCCGGATCTGCCCTATTTAT	23
BSP F	AGGCATAAACGGCACCAGTACCA	23
BSP R	CTTGCCCTGCCTTCCGGTCT	20
BGLAP F	GGAGGGCAGCGAGGTAGTGAAGA	23
BGLAP R	GCCTCCTGAAAGCCGATGTGGT	22
RUNX2 F	CCGCCATGCACCACCACCT	19
RUNX2 R	CTGGGCCACTGCTGAGGAATTT	22
Integrin F	TGGGCGCTACTGTCATTTGGG	21
Integrin R	CTGGCATCGGGTAGCTAGAGGC	22
ALP F	GATGTGGAGTATGAGAGTGACG	22
ALP R	GGTCAAGGGTCAGGAGTTC	19
Osteonectin F	TTGCAATGGGCCACATACCT	20
Osteonectin R	GGGCCAATCTCTCCTACTGC	20
