# Supplementary material for: Incorporation of Fucoidan in β-Tricalcium phosphate-Chitosan scaffold prompts the differentiation of human bone marrow stromal cells into osteogenic lineage
Source: Sci Rep. 2016 Apr 12;6:24202. doi: 10.1038/srep24202 (PMC4828646; doi:10.1038/srep24202)
Supplement: Supplementary Information [file srep24202-s1.docx]

Incorporation of Fucoidan in β-Tricalcium phosphate-Chitosan scaffold prompts the differentiation of human bone marrow stromal cells into osteogenic lineage

^1^Subramaniam Puvaneswary, ^1^Hanumantharao Balaji raghavendran ^2^Sepehr Talebian, , ^1^Malliga Raman Murali, Suhaeb AM, Simmrat Singh, ^1^Tunku Kamarul

^1^Tissue Engineering Group (TEG), Department of Orthopaedic Surgery, NOCERAL, Faculty of Medicine, University of Malaya, 50603, Kuala Lumpur, Malaysia

^2^Department of Mechanical engineering, Engineering Faculty, University of Malaya, 50603 Kuala Lumpur, Malaysia

Characterization of bone marrow stromal cells based on surface marker using flowcytometry


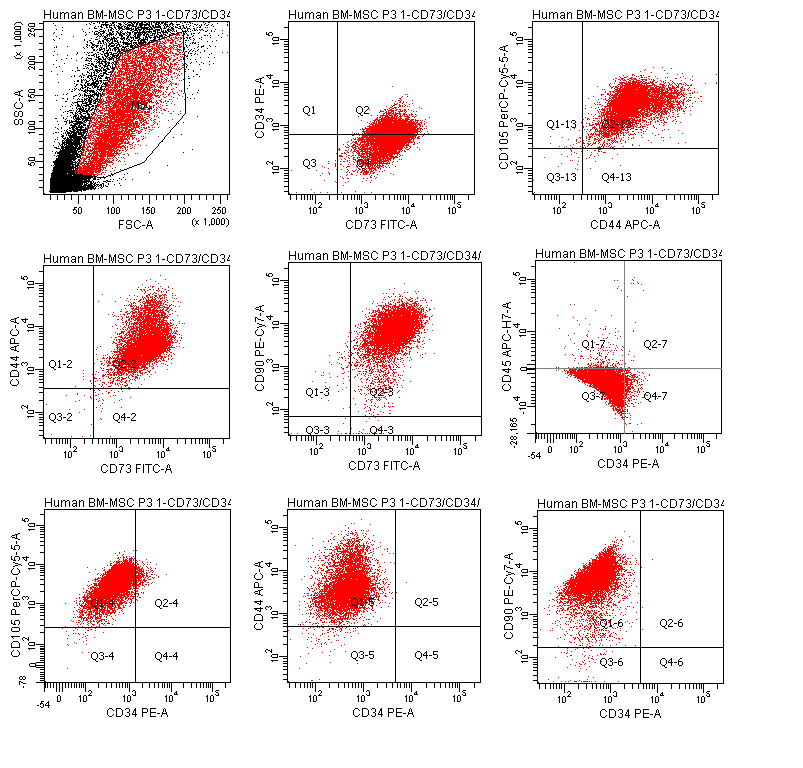


**Flow cytometry analysis**

Adherent stromal cells (1X10^6^)/ml were trypsinized at P3. Cells were transferred into 15-mL conical tubes and centrifuged at 400g for 5 minutes. The supernatant was discarded and pellet was resuspended in culture medium. This was centrifuged again at 400g for 5 minutes and the supernatant was discarded. The cells were resupended in 100 µl of 1X PBS and aspirated up and down through a pipette several times to help disaggregate clumps. 0.5 X 10^6^ cells was transferred into 12 X 75 mm falcon polystyrene FACS tube and stained with appropriate amount of conjugated antibodies.

The quantity of each antibody conjugated with fluorochromes was added to the cells in each tube (10µl of CD34-PE, 5µl of CD90- PER CP CY7, 5µl of CD 105- PER CP 5.5, 5µl of CD 45- APC H7, 5µl of CD 73 – FITC, 10µl of CD 44- APC) respectively. All tubes were incubated for 20 minutes in dark with ice. After incubation, cells were washed in 2 ml of phosphate buffer saline and centrifuged at 300g for 5 minutes, to remove the unbound antibodies. The pellet was further resuspended to 500µl with 1x PBS. Data was analyzed and acquisition was performed using FACSDIVA Software, Becton Dickinson. Flow cytometer instruments were set using unstained cells. Cells were gated by forward versus side scatter to eliminate debris. The number of cells staining positive for a given marker was determined by the percentage of cells present within the established gate. A minimum of 10,000 events was characterized and recorded.

| Surface biomarker | Results | % of cells |
| --- | --- | --- |
| CD44 | + | 98.5 |
| CD73 | + | 63.3 |
| CD90 | + | 98.8 |
| CD105 | + | 95.7 |
| CD34 | - | 36.4 |
| CD45 | - | 2.2 |
